# Colour variation in cichlid fish: Developmental mechanisms, selective pressures and evolutionary consequences^☆^

**DOI:** 10.1016/j.semcdb.2013.05.003

**Published:** 2013-06

**Authors:** Martine E. Maan, Kristina M. Sefc

**Affiliations:** a University of Groningen, Behavioural Biology, PO Box 11103, 9700 CC Groningen, The Netherlands; b Institute of Zoology, University of Graz, Universitätsplatz 2, A-8010 Graz, Austria

**Keywords:** Cichlidae, Natural selection, Pigmentation, Polymorphism, Sexual selection, Speciation

## Abstract

Cichlid fishes constitute one of the most species-rich families of vertebrates. In addition to complex social behaviour and morphological versatility, they are characterised by extensive diversity in colouration, both within and between species. Here, we review the cellular and molecular mechanisms underlying colour variation in this group and the selective pressures responsible for the observed variation. We specifically address the evidence for the hypothesis that divergence in colouration is associated with the evolution of reproductive isolation between lineages. While we conclude that cichlid colours are excellent models for understanding the role of animal communication in species divergence, we also identify taxonomic and methodological biases in the current research effort. We suggest that the integration of genomic approaches with ecological and behavioural studies, across the entire cichlid family and beyond it, will contribute to the utility of the cichlid model system for understanding the evolution of biological diversity.

## 1 Introduction

Cichlid fishes are well-known among aquarists and biologists for their enormous colour diversity. The family comprises between 2000 and 3000 species that inhabit rivers and lakes in tropical and subtropical regions of Africa and the Americas, as well as India and Sri Lanka. Taxonomically, Cichlidae are divided into several tribes, among which for example the African Haplochromini are renowned as particularly species rich and colourful. It is not only their phenotypic diversity that makes cichlids so fascinating, but also the speed at which some of this diversity evolved. For example, the several hundred species of the East African Great Lakes emerged within tens of thousands to some million years. Closely related species often differ in little else but the colour of body and fins.

In many species, body colours are overlaid with dark vertical bars and/or horizontal stripes. Frequently, the differently coloured body regions are not defined by sharp boundaries but rather shade into one another—in contrast to the sharp-edged patterns of many well-known coral reef fishes.

Colour patterns vary not only between cichlid species, but also within and among populations of a species (sexual dichromatism, polychromatism and geographic variation), as well as within individuals, depending on their age and social status.

In this review, we aim to provide an overview of this variation, its underlying mechanisms and evolutionary consequences, and identify knowledge gaps and priorities for future research.

## 2 Colour pattern variation in the cichlid fish family

### 2.1 Variation within populations

Polychromatism, i.e. colour pattern variation within populations, occurs both as sexual dichromatism and sex-independent variation. The widespread dichromatism with conspicuous male and more cryptic female colouration is traditionally attributed to sexual selection on males of polygamous species with maternal brood care [1]. In some monogamous cichlid species, the function of body colours in social communication may preclude dichromatism, as both sexes rely on colour or patterns to meet sex-independent social challenges such as territory defence and individual recognition [2–5].

Examples of intrasexual polychromatism include yellow or blue fin colouration in males of several Lake Tanganyika cichlids, female-linked blotch polymorphisms in several haplochromine species of Lakes Malawi and Victoria, and sex-independent grey-black or gold morphs in the Midas cichlid species complex. In a few instances, colour assortative mating has been observed, and what is currently regarded as polychromatism may in some cases represent the initial stages of sympatric speciation [6,7].

### 2.2 Geographic variation

**Fig. 1:**
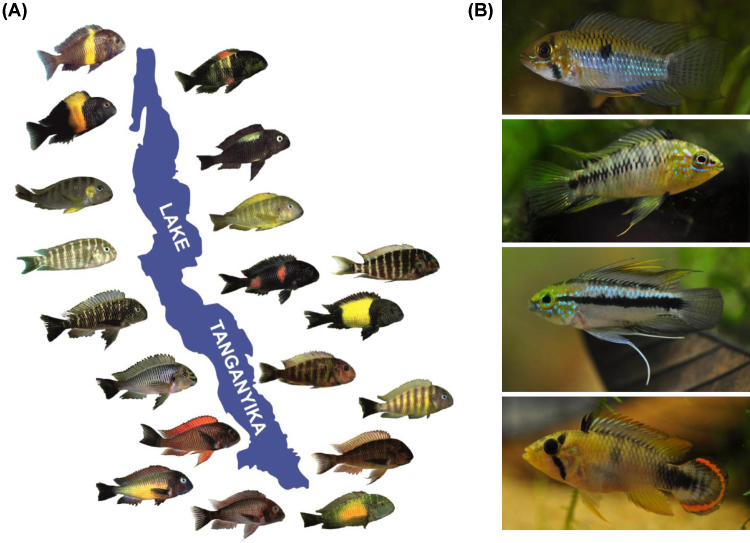
Colour variation in African and neotropical cichlids. (A) *Tropheus* spp. colour morphs in Lake Tanganyika (photos by Wolfgang Gessl: www.pisces.at and Peter Berger: www.afrika-cichliden.de; see also Egger et al. [93]). (B) *Apistogramma* spp from the Amazon basin (top to bottom: *A. steindachneri*, *A. borelli*, *A. trifasciata*, *A. panduro*. Photos by Ricardo Britzke).

Many cichlid species display geographic variation in colour patterns, which can range from rather subtle differences in hue and patterning to conspicuous differences in both colour and pattern (Fig. 1). This variation can interfere with species delimitations, particularly when sexual isolation by mate choice and postzygotic isolation due to genetic incompatibilities are not tested. For example, the taxonomic treatment of geographically variable taxa has not been consistent across the three East African Great Lakes Malawi, Victoria and Tanganyika, and a level of variation considered intraspecific in one lake may correspond to allopatric species in another lake [8]. Notwithstanding the many unresolved taxonomic problems haunting cichlid scientists, it is clear that geographic isolation contributes critically to the evolution and preservation of phenotypic variation in both riverine and lacustrine cichlids [9–11]. In lakes, species in the structured littoral tend to be more variable in colouration than pelagic or deep-water demersal species [12–14].

Population genetic studies in African lake cichlids confirm that populations of stenotopic species, i.e. those with a narrow specialisation to certain environmental conditions, are often isolated from each other, even by rather small habitat barriers (e.g., [15–19]). While geographic colour pattern differentiation is usually accompanied by genetic differentiation, not all species with distinct population structure exhibit phenotypic variation (e.g. [18,20]). Geographic isolation certainly facilitates and maintains phenotypic differentiation, but other factors such as genetic predisposition, environmental variation and mate preferences may play a crucial role in the evolution of allopatric phenotypic differentiation. In several Lake Victoria cichlids, colouration covaries with water transparency among populations, suggesting an environmental component to geographic colour pattern variation [21–24]. In many cases, however, the ecological significance of colour pattern differentiation remains unclear (e.g. [25]).

Colour variation within and among cichlid species often involves the convergent evolution of similar patterns, e.g. the repeated occurrence of similar colour combinations in distantly related species of South American *Crenicichla* lineages [9] and Lake Malawi haplochromines [16,26], repeated shifts between two patterns of gill-cover markings within a species of the Lake Tanganyika Lamprologini [2], and the widespread occurrence of a blotch polymorphism in both Malawi and Victoria haplochromines [27]. Studies into the genetic basis of these parallel patterns, however, tend to identify different underlying genetic factors ([28], but see [29]).

## 3 Cellular and molecular basis of cichlid colouration and colour pattern differentiation

### 3.1 Pigments and structural colours

Vertebrate body colours and patterns are determined by the distribution, density and aggregation state of different chromatophores in the integument. In teleost fish, colours are produced by light absorption of pigments contained in the chromatosomes of melanophores (containing black eumelanin pigment), erythrophores and xanthophores (with yellow-red carotenoid and pteridine pigments) and cyanophores (containing blue pigment of unknown chemical composition), as well as by reflection from purine crystals, which, depending on their spatial organisation in refractosomes or leucosomes, produce the metallic iridescence of iridophores or the whitish hue of leucophores (e.g. [30–32]). Fish can de novo synthesise eumelanin from tyrosine and pteridine pigments from GTP, whereas carotenoids have to be supplied by the diet. Chromatophores specific for these three pigment types as well as iridophores occur in the skin, scales and fins of cichlid fish (e.g., [29,33,34]). While in other freshwater fish species, UV reflectance can be an important component of nuptial colouration (e.g. [35,36]), and several cichlids can perceive UV light (e.g. [37]), little is known about the role of UV in cichlid visual communication [38].

### 3.2 The genetics of cichlid colouration

#### 3.2.1 Genetic architecture of colour patterns

Although diverse and complex genetic and cellular processes are involved in the differentiation and distribution of chromatophores and the synthesis and metabolisation of pigments in teleost fish (e.g. [39]), estimates of the number of genetic factors responsible for differences between cichlid colour pattern traits consistently range between one and seven, whether they are derived from phenotypic segregation, QTL mapping or genomic screens based on population genetic principles, or gene expression assays [25,34,40–44]. These numbers may be underestimates for reasons of analytical power and assumptions of the statistical methods, but they suggest that only few genetic changes may be required to effectuate colour pattern differentiation. Furthermore, inheritance patterns of colour traits suggest dominant gene action at many of the putative colour loci [34,40,41].

#### 3.2.2 Modularity and integration

In the cichlid radiations of Lakes Malawi and Victoria, male nuptial colour pattern variation often derives from different combinations of core modules such as blue or yellow/red body, blue or yellow/red ventrum, blue or yellow/red dorsum, and the presence or absence of dark vertical bars or horizontal stripes [26,34,45]. Analogous transitions between colour traits occurred repeatedly in different species pairs, and similar trait combinations can be found in distantly related taxa [26]. Correlations among colour pattern traits, e.g. colours of the individual fins, in segregating F2 populations suggest that some of these modules are not expressed independently but phenotypically integrated [34,46]. Modularity and integration have opposite effects: while modularity allows the assembly of colour traits in different combinations and can hence promote the rapid evolution of novel patterns, the integration of modules constrains the possible combinations and forces certain phenotypic changes to coincide. Particularly in haplochromine cichlids, the rapid divergence of conspicuous male colour patterns contrasts with slow and small changes in the females’ rather drab colouration. Certainly, male and female colour patterns are subject to different selection regimes, favouring showiness in one sex and crypsis in the other. A resolution to the resulting sexual conflict has recently been identified at the level of sex-specific colour trait integration. Colour traits are less integrated in males than in females, which facilitates the flexible combination of core colour modules into various male nuptial colour patterns, while restraining the diversification of female colouration [46].

### 3.3 Molecular processes promoting phenotypic diversification

#### 3.3.1 Phenotype evolution associated with gene expression regulation

**Fig. 2:**
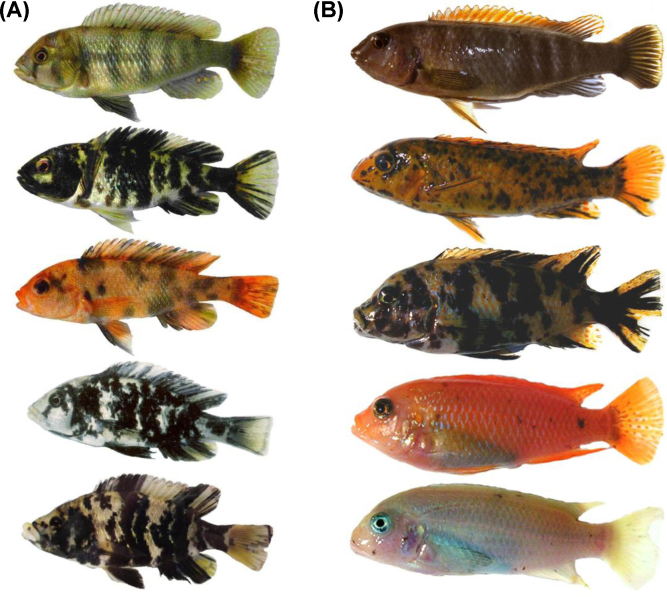
Examples of the Haplochromine blotch polymorphism (all females). (A) Lake Victoria, from top: *Neochromis omnicaeruleus*: ancestral brown phenotype (P morph), orange blotched (OB morph), white blotched (WB morph); *Paralabidochromis chromogynos* (WB morph); *P. chilotes* (WB morph). *Paralabidochromis* photos by Ole Seehausen. (B) Lake Malawi, from top: *Labeotropheus trewavasae* (P morph); *L. trewavasae* (OB morph); *Metriaclima xanstomachus* (OB morph); *M. pyrsonotus* (OB morph, commonly called ‘orange’ morph); *M. callainos* (OB morph, commonly called ‘white’ morph). All of the morphs presented are heterozygous for the OB allele of *pax7*, regardless of degree of blotching. (Malawi photos by Reade Roberts).

Whereas cichlid species show little genetic variability in protein coding sequences in relation to their eminent phenotypic diversity, gene expression differences, diversity in untranslated mRNA regions with potential regulatory effects, as well as cis- and trans-regulatory mutations highlight the importance of regulatory factors in the evolution of cichlid diversity [42,47–52]. In particular, trans-regulatory mechanisms may allow coordinated phenotypic changes, and independent mutations in one particular regulator gene might underlie the emergence of similar colour patterns in different cichlid taxa. Alternatively, the expression of a particular colour pattern phenotype may require a specific combination of alleles segregating at interacting regulatory loci, such that, whenever this condition is fulfilled, the integrated phenotype emerges in a phenotypically monomorphic, but genetically polymorphic, population [45]. Moreover, colour pattern polymorphisms can be shared between related species by way of ancestral polymorphism or gene flow. For example, a blotch polymorphism (orange blotch [OB] and white blotch [WB], Fig. 2) occurs in several species from Lake Malawi and Victoria and is linked to a female sex determining locus, and therefore largely restricted to females [27]. Blotched females display dark melanophore blotches of variable size and number on orange or white background, and occur alongside the ‘normal’ brown barred females. Recently, comprehensive QTL and association mapping studies [43,53] corroborated the localisation of the Lake Malawi OB polymorphism [54] and determined that a cis-regulatory mutation in the *pax7* gene, a transcription factor involved in the development of pigment cell lineages from neural crest precursors, underlies the blotch phenotype in all tested species of the Lake Malawi flock. Blotched females have higher *pax7* expression than brown barred females, and the phenotypic effect of the upregulation–fewer but larger melanophores—corresponds to the effect of *pax3* in zebrafish [53]. A Lake Victoria OB individual examined for the *pax7* polymorphism showed the ancestral, ‘brown-barred’ haplotype, indicating a different genetic basis and thus an independent origin of the Lake Victoria blotch phenotype [53].

In addition to the *pax7* polymorphism, regulatory changes have also been inferred in other studies of cichlid pigmentation and colour pattern evolution. The colony-stimulating factor 1 receptor *csf1r*, coding for a receptor tyrosine kinase essential for recruiting xanthophores from precursor cells, is expressed in the yellow ‘egg spot’ markings found on the anal fins of males of the tribe Haplochromini (see Box 1), but not in the tissue surrounding the egg spots [29]. A TATA-box mutation upstream of *csf1r* was found to be specific to the haplochromine lineage, in which these egg spots evolved, and is a candidate locus for the regulation of egg spot development. Furthermore, the accelerated evolution of the ligand-binding domains of the haplochromine *csf1r* in comparison to basal cichlid lineages lacking egg spots suggested an association between coding sequence mutations in the *csf1r* gene and egg spot evolution [29]. Interestingly, *csf1r* was also expressed in the yellow spots on the elongated pelvic fins of *Ophtalmotilapia ventralis* males, indicating either a general function in xanthophore development, or a specific but shared genetic pathway despite independent evolutionary origin [29]. Egg spots and other pigment containing tissues of the cichlid *Astatotilapia burtoni* express a gene of the endothelin family (*edn3b*; as well as its receptor, *ednrB1a*; [55]) known to be involved in vertebrate pigmentation. Across African cichlid species, amino acid changes occurred mainly in the precursor part of the Edn3b protein, which might affect its posttranslational regulation, and in parts of the EdnrB1a receptor protein conferring ligand binding selectivity, which could affect ligand-receptor binding functions [55]. A comparative gene expression study addressing the yellow/blue sexual dichromatism of *Pseudotropheus saulosi* (a Malawi haplochromine) identified differences in expression levels of *copz-1*, a gene coding for a subunit of a protein complex involved in the endosomal-to-Golgi vesicle trafficking pathway [42].

**Fig. 3:**
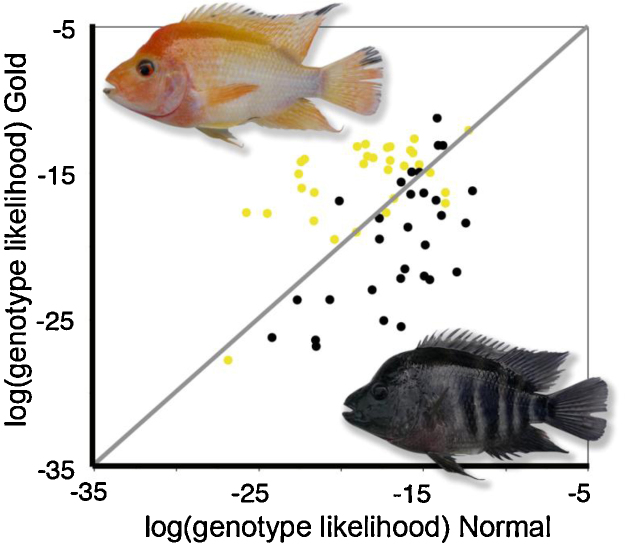
Genetic assignment of *Amphilophus xiloaensis* gold and normal colour morphs (Midas cichlid species complex) from Lake Xiloa, Nicaragua. Dots (black for normal, yellow for gold) represent the likelihood that a particular individual originates from a given colour morph sample. Overall, more than 80% of individuals were correctly assigned, based on microsatellite allele frequencies. These results suggest that colour morphs mate assortatively, consistent with observations of territorial pairs in the lake.

A sex-independent dark/gold colour polymorphism occurs in several species of the Midas cichlid species complex inhabiting crater lakes in Nicaragua (named after the legendary King Midas, who had everything he touched turn into gold [56], Fig. 3). The gold phenotype emerges in a small proportion of adults when the melanophores causing the dark juvenile phenotype die and the underlying xanthophores become visible [57]. A single dominant mutation determines the gold phenotype, but in contrast to melanic colour polymorphisms in tetrapod vertebrates, which are often caused by mutations in the coding sequence of the transmembrane melanocortin receptor 1 gene [58,59], no *mc1r* sequence polymorphism was found to be linked to colouration in the Midas cichlids [60]. Contradictory with its function in melanin synthesis, *mc1r* was upregulated in the gold morph, perhaps as a side effect of the genetic changes underlying melanophore loss [60].

#### 3.3.2 Gene duplications

Body colour patterns and pigment cell types are more diverse in fishes than in any other vertebrate lineage, and it has been suggested that the evolution of this diversity was promoted by genome duplications, in particular a fish-specific genome duplication (FSGD) at the base of the teleost lineage, which increased the repertoire of genes potentially involved in pigmentation [39].

Following a duplication, species- or lineage-specific loss of gene duplicates, acquisition of novel functions by one paralog (neofunctionalisation) and the division of the original function between paralogs (subfunctionalisation) can contribute to the evolution of novel phenotypes. For example, tissue-specific expression of the paralogs can mitigate adverse pleiotropic effects of colour gene mutations on other traits [39], and life stage-specific expression of paralogs [61] can allow a trait to evolve even if its function in a particular life stage would otherwise constrain variation.

Indeed, whereas duplicated genes are typically lost from the genome at high rates, the majority of genes known to be involved in melanin and pteridine colour patterns have been retained in duplicate after the FSGD [39]. Duplicated colour genes cloned in cichlids include the receptor tyrosine kinase *csf1r* paralogs originating from the FSGD, of which the A paralog retained its function in cichlid pigmentation [29] whereas the B paralog may have functionally diverged [62]. Furthermore, one gene of the endothelin family, which expanded via whole genome duplications, is associated with cichlid colouration [55]. Surprisingly, none of the two cichlid-specific paralogs of a gene responsible for a zebrafish colour pattern mutant (*ci-kir7*) are expressed in the cichlid integument [63].

#### 3.3.3 Alternative splicing

Alternative splicing is a mechanism by which different mRNAs are produced from the same gene, e.g. by exon skipping or intron retention, thus increasing the coding capacity of the genome and enhancing phenotypic diversity [64]. The *hagoromo* gene regulates a developmental pathway essential for the formation of colour patterns via protein degradation and is associated with a stripe pattern mutant in the zebrafish. In a survey across African cichlids comprising both old and recently radiated lineages, a total of nine different splicing variants (mRNA species) were detected. Splicing patterns consisted of two to nine mRNA variants per individual, were species-specific and consistent within species, and therefore might be linked to species-specific colour pattern characteristics. Furthermore, splicing complexity was correlated with speciation rate [65]. Since different mRNA species can regulate each other's translation, and the eventual combination of *hagoromo* proteins may determine the range of possible targets for protein-protein interactions associated with colour pattern development, it is conceivable that the propensity to evolve novel colour patterns is linked to the number of *hagoromo* mRNA species available for reshuffling.

## 4 Developmental and environmental plasticity

**Fig. 4:**
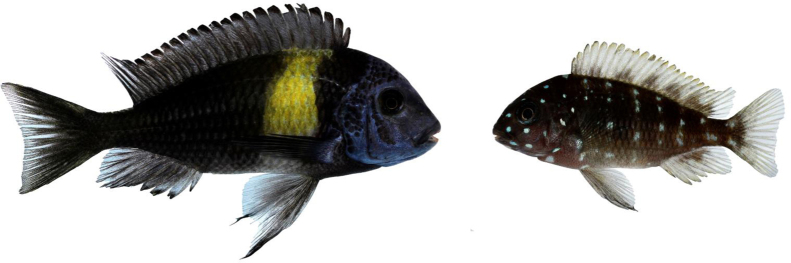
Adult and juvenile colour patterns of *Tropheus duboisi* (Lake Tanganyika, Maswa). Photos by Wolfgang Gessl (www.pisces.at).

Colour pattern changes during ontogeny, e.g. upon maturation, typically involve morphological modifications in pigment concentration and in the density and distribution of chromatophores [32,57]. These changes are quite dramatic in some cichlid species, e.g. from a typical juvenile pattern of pale and dark bars to the species-specific colourful adult patterns, or from a bright yellow juvenile to dark adult colouration (Fig. 4). Moreover, adults of many species change between breeding and non-breeding colours (e.g. [3]).

Social and other environmental factors demand plasticity and versatility in colouration and patterning within a given developmental stage. Rapid physiological changes in colours and patterns, effected by dispersal or aggregation of pigment vesicles under neural or hormonal control, are involved in signalling motivational or reproductive state (e.g., [5,66,67]). During agonistic encounters, colour pattern switches may reduce escalation by coordinating the exchange of information between opponents [68]. Subdominant individuals often become darker (e.g. [69–71]), sometimes reducing aggression by the dominant opponent (*Astronotus ocellatus* [72]). In convict cichlids (*Cichlasoma nigrofasciatus*), amelanistic males lack the ability to modulate colouration, which may explain their competitive disadvantage against wildtype (barred) males [73].

Over somewhat longer timescales (i.e. days or weeks), colouration may develop in a social status-dependent manner. In addition to the common phenomenon that colour expression depends on reproductive maturation and activity, several haplochromine species exhibit transitions between yellow and blue phenotypes [74–76]. These colour variations, just like colour differences between closely related species, can be associated with morph- or species-specific steroid and behavioural profiles [77–80]. Little is known, however, about the underlying mechanisms, and no causal relationships have been established.

In the haplochromine cichlid *Astatotilapia burtoni*, shifts in the males’ social status between territoriality (dominance) and non-territoriality (subordination), which can occur repeatedly during an individual's lifespan, are accompanied by changes in size, reproductive capacity, hormone levels, behaviour, and colour pattern, with both morphological and physiological changes involved in the colour pattern transformations [81]. Additionally, territorial males can change reversibly between bright blue and bright yellow overall body colour, and the dominance of yellow over blue males suggests that this plastic colour polymorphism serves as a social signal [74,77].

Colouration can also be adjusted plastically to habitat characteristics, for example to convey camouflage against a certain background. In the lamprologine *Telmatochromis temporalis*, pale and dark coloured individuals occupy well-lit and shaded territories, respectively, and can reverse their colouration within weeks following transplantation between habitat types [82].

## 5 Evolution

### 5.1 Drift and hybridisation

Cichlid body colours play important roles in social communication, competition, mate choice, predation and foraging, and are therefore subject to multiple potentially strong selection pressures. At the same time, high levels of population structure, which are typical e.g. for rock-dwelling cichlids of the African Great Lakes, open a path for phenotypic differentiation by genetic drift. Indeed, when it was found that even minor habitat barriers prevented gene flow between populations of stenotopic littoral cichlids, it was immediately suggested that drift could be involved in allopatric diversification [15,83,84]. So far, however, only few studies tested the evidence for selection against a null hypothesis of random drift. In a gene involved in xanthophore development (*csf1r-a*), adaptive sequence evolution under positive selection was inferred from the ratio of nonsynonymous to synonymous substitutions, and was related to the emergence of the egg spot pattern displayed on the anal fins of haplochromine cichlids [29]. A different approach, employing genomic scans of neutral population differentiation with anonymous multi-locus markers, demonstrated that drift could not be the sole cause of allopatric colour pattern differentiation among morphs of the genus *Tropheus* [25]. While drift is highly likely to influence the phenotypic evolution of fragmented cichlid populations, it may prove very difficult to identify examples in which selection can be considered unimportant, and likewise challenging to quantitatively assess the relative contributions of drift and selection to phenotypic differentiation.

The combination of population fragmentation and habitat change, such as when geomorphic instabilities shift entire rivers or when water level fluctuations reshape lake shores, provides opportunities for secondary contact and hybridisation among phenotypically differentiated populations. Moreover, ancient polymorphisms are shared among African cichlids across large geographic distances [85,86]. Possibly, riverine species act as transporters of genetic variation between lake species assemblages [86]. It is becoming increasingly clear that the evolutionary histories of various cichlid lineages are affected extensively by (ancient) hybridisation (e.g., [85–88]). The consequences of hybridisation on phenotype evolution range widely from the erosion of phenotypic diversity [21] to the wholesale promotion of adaptive radiations [85,86,89,90]. With regard to cichlid colouration, experimental crosses have demonstrated the potential of hybridisation to create novel colour patterns (e.g. [34,91]), and in a few instances, naturally occurring colour morphs show signatures of a hybrid origin [92,93].

### 5.2 Natural selection

#### 5.2.1 Linking melanin patterns with ecology and social behaviour

In African cichlids, the expression of either bars or stripes is correlated with habitat type: vertical bars predominate in littoral cichlids living in rocky or vegetated habitats, where barred patterns may improve camouflage against the structured background [45]. Furthermore, many littoral cichlids are highly territorial, and since vertical bars are often associated with aggression in cichlids [3], competition for territories in the littoral may contribute to the correlation [45]. In contrast, horizontal stripes are common among cichlids aggregating in shoals. These are signals of social inferiority and may reduce aggression among shoaling individuals. They are typical for piscivorous cichlids as well, possibly hampering the visual perception of the piscivore by its prey [45]. These correlations, however, are not without exceptions. For example, the pelagic piscivorous species in the tribe Bathybatini show both barred and striped patterns, and several territorial, littoral rock-dwellers, e.g. in the tribe Lamprologini, have longitudinal stripes. Whether or not these exceptions are due to additional selective factors, as opposed to non-adaptive genetic constraints, remains unknown.

#### 5.2.2 Predation risk

Body colouration can dramatically affect visual predation. In the African Lakes, diurnal predators on adult cichlids include other fishes (e.g. catfish, lungfish, Lates species, piscivorous cichlids), reptiles (crocodiles, snakes) and birds (cormorants, pelicans, herons, kingfishers, birds of prey), and similar predation pressure is faced by cichlids in other regions. The guppy *Poecilia reticulata* famously illustrates how the evolution of colouration is closely linked to predation pressure [94]. In cichlids however, while many researchers have speculated about colour-dependent predation, data are largely lacking. The only experimental study that we know of tested for differences in predation risk between three colour morphs of the Lake Victoria haplochromine *Neochromis omnicaeruleus* and found that blotched morphs, particularly orange-blotched (OB), were attacked more often by pied kingfishers [95]. These results are in contrast with the widely held assumption that the blotched phenotype actually provides camouflage [53,96,97]–an idea that could help to explain why blotch is sex-linked, as camouflage would be favoured in females but sexually selected against in males [53]. The discrepancy may be due to the large variation in the appearance of different blotched morphs, both within and between species (Figure 2): blotched colouration ranges from all-orange to almost completely black, with many intermediate phenotypes that have a peppery colouration or much larger black patches on a white, yellowish, orange or pink background. Clearly, the effects of colouration on predation risk represent a major knowledge gap in cichlid research.

#### 5.2.3 Foraging

On the other end of the predator-prey interaction, body colouration affects the hunting efficiency of predators, and several cichlid species have evolved adaptive colour patterns to facilitate their access to prey. Lepidophagous cichlids feed on fish scales which they pick from the bodies of other fish, and mimicry of their prey species may allow several of these scale-eating cichlid species to mingle with their targets prior to attack [98,99].

Mimicry and crypsis may be linked to the sexual colour polymorphism in the scale-eater *Plecodus straeleni*, in which the cryptic beige females attack sand-dwelling species near the substrate, whereas the striped males forage in the water column; their conspicuous pattern may mimic that of likewise striped, but harmless species. Whether this mimicry serves to gain access to the model itself, or, disguised as the harmless models, to other fish species, remains unclear [100]. Similarly, the sex-independent dark-pale dichromatism of several predatory cichlids correlates with their hunting behaviours, with dark individuals hunting in the space under rocks and pale individuals in open water [101].

Even in non-predatory species, colouration can affect access to food. The zoobenthivorous *Neolamprologus mustax* forages preferentially in the territories of a particular algivorous host species and, in some regions, mimics the yellow body colour of the host's juveniles. Experiments suggest that by displaying the host's juvenile colour, the guest species may reduce aggression of the highly territorial hosts and gain admittance to their territories [102].

## 6 Intersexual selection: colouration-mediated mate choice

Cichlids are frequently found to mate assortatively by colour or melanin pattern, both in nature [7,103–105] and in experimental settings (Table 1). In full contact trials, sexual isolation between species or allopatric morphs increases with colour pattern dissimilarity in some cichlids (e.g. [106–109]), but many other traits may influence mate choice (e.g. acoustic cues [110–112], olfactory cues [113,114], and male territory characteristics [115–120]. The number of studies addressing the role of colouration in mate choice while excluding non-visual modalities is rather small and limited to a few species (Table 1). Moreover, research on colouration-mediated mate choice is taxonomically biased, with the majority of studies focusing on African lake cichlids. Yet, several studies in neotropical species suggest similar patterns. The species-rich South American genus *Apistogramma* harbours extensive (male) colour diversity between species and allopatric populations (Figure 1). Allopatric colour morphs of *A. caetei* mate assortatively in the laboratory [121]. In *A. cacatuoides*, there is geographic variation in both female preferences and male colouration [108,122].

Also within populations, colour polymorphisms may coincide with non-random mating. In the blotch-polymorphic Lake Victoria cichlid *Neochromis omnicaeruleus* (Figure 2), there are significant (but incomplete) assortative male preferences for female colour morph [123,124] see also [125], possibly leading to nonrandom mating in the wild [126]. However, the scarcity of blotched males in natural populations in nearly all species in which the polymorphism occurs [27] limits the opportunity for phenotype-assortative mating. Another fairly well-studied example is the ‘gold’ polymorphism that occurs in several cichlid species in Nicaraguan lakes. Morph-assortative mating, albeit incomplete, has been observed in several species of Midas cichlids [7,127], sometimes leading to significant genetic differentiation ([7,128,129]; Fig. 3).

Taking together the experimental evidence, we may conclude that cichlid colouration often affects population- or species-assortative mate choice—but not always (e.g. [114,130]). More work is required to establish the relative importance of visual and other cues, and to identify taxonomic patterns.

## 7 Sexual selection, colour divergence and speciation

Dominey [131] was among the first to suggest that sexual selection on male colouration may be involved in the speciation of African cichlids. This hypothesis inspired a multitude of studies that found support for colour-mediated assortative mating, some of which were mentioned above.

In addition, several comparative analyses identified associations between sexual selection, colour variation and species divergence. Seehausen et al. [45] found that among East-African cichlids with promiscuous mating systems, in which sexual selection is expected to be strong, sister species often differ in male nuptial hue. A few years later, Allender et al. [26] documented extreme evolutionary lability of Lake Malawi cichlid colouration, characterised by repeated and parallel evolution of colour patterns across genera. Most recently, Wagner et al. [132] showed that African lake cichlids are more likely to radiate into multiple species if they are sexually dichromatic, again supporting the idea that sexual selection on colouration promotes cichlid speciation.

### 7.1 Intraspecific sexual selection and interspecific isolation

Fisherian sexual selection may amplify initially small differences in genetic variation between populations [133,134]. Speciation by divergent sexual selection on colouration would imply that colouration not only mediates species-assortative mating, but is also subject to directional sexual selection *within* species. Cichlid mating systems are diverse (e.g. [3,4,135]) and sexual selection is expected to be stronger in some lineages than others. In particular, polygynous mating systems and female-only parental care set the stage for potentially strong sexual selection by female choice. But does such intraspecific choosiness target the same colouration traits that also determine assortative matingtd:quest In the Lake Victoria haplochromine *Pundamilia nyererei* it does: females prefer redder males, and red colouration is also the main cue determining species-assortative preferences [115]. In the Malawi haplochromine *Labeotropheus fuelleborni*, male colouration is also subject to intraspecific female choice, but the colour traits that females find most attractive within populations do not mediate preferences between populations [136]. Together, these studies suggest that intraspecific sexual selection on haplochromine colouration might be common, but more species need to be investigated in order to draw general conclusions.

There are some data from other lineages suggestive of within-population or within-species sexual selection for colouration traits. We already mentioned the colourful *Apistogramma* genus, in which allopatric variation in male colouration coincides with variation in female preferences, possibly indicating directional sexual selection towards population-specific colour phenotypes [108,121,122]. This resembles observations in *P. nyererei* of allopatric variation in both male red colouration and the strength of female preference for this trait [22].

Although similarities and discrepancies between inter- and intraspecific mate choice are important for inferring the role of sexual selection in trait divergence and speciation, very few studies investigate mate choice in both these contexts. We suggest that the extensive diversity in cichlid colours should inspire more studies into colouration-mediated mate choice along the entire continuum of populations, morphs, and species.

### 7.2 Ecological correlates of sexually selected colour variation

In addition to divergent Fisherian runaway selection, divergence in sexually selected colouration may be more deterministic, driven by consistent differences in ecological conditions. One scenario that has received considerable attention in recent years is sensory drive: the evolution of sexual communication traits in response to heterogeneous signalling conditions [137]. If cichlid colours evolve to maximise conspicuousness to potential mates, they are expected to adapt to local light regimes, as has been observed in other fish species [138–141]. Evidence for such adaptation mainly comes from the haplochromines from lakes Malawi and Victoria.

**Fig. 5:**
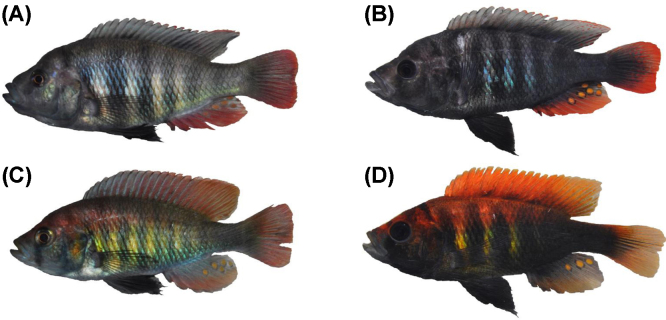
The Lake Victoria species pair *Pundamilia pundamilia* (A and B) and *Pundamilia nyererei* (C and D). Fish on the left (A and C) are from Python island, where the water is relatively turbid and hybridisation sometimes occurs. The more distinctly coloured fish on the right (B and D) are from Makobe island, where waters are clear and no hybridisation is observed.

For example, male colouration becomes more saturated with increasing water transparency in some Lake Victoria haplochromines ([21,23]; Fig. 5), and the predominance of blue and yellow colouration patterns in Lake Malawi may be a consequence of a light regime that maximises conspicuousness of these specific hues [26,142].

Likewise, species-specific monochromatic patterning in the deeper waters of Lake Malawi, where the narrow light spectrum precludes hue discrimination, is consistent with predictions of sensory drive [103]. However, a systematic analysis of associations between light environments and male nuptial colouration, in haplochromines and other lineages, is lacking (but see, for Lake Malawi: [143–145]).

For explaining speciation, divergent adaptation of colouration represents only part of the story. A stronger case for divergent sensory drive could be made if differences in sensory environments generate changes in visual system properties, such that visually guided mate choice would select for different colours in different environments. Recent work in both lakes indeed indicates that colour vision properties are highly diverse among cichlid species and populations (e.g. [61,146,147]). In the well-studied species pair from Lake Victoria *P. pundamilia* and *P. nyererei*, differences in retinal visual pigments are correlated with underwater light regimes as well as male nuptial colouration, such that the red-preferring *P. nyererei* females inhabit deeper, red-shifted waters, and the extent of visual pigment differentiation predicts the strength of reproductive isolation [148]. But whether or not red-shifted colour vision is mechanistically involved in the development of mating preferences for red males remains to be tested [149]. Lake Malawi haplochromines are much more diverse in colour vision but species differences show fairly little association with either visual environments or nuptial colouration [147,150]. For non-haplochromine lineages, little is known about the effects of variation in underwater visual environments on the evolution of colour patterns and colour vision.

Additional sources of environmental heterogeneity may affect colouration traits. As noted above, predators may exert significant selective pressure on cichlid colouration, and their abundance and species identity undoubtedly vary between geographic areas as well as depth ranges. Likewise, colours that depend on dietary pigments may covary with the availability of such compounds in the environment. The idea that these environmental variables may drive divergence in nuptial colouration are commonly addressed in other taxa (e.g. guppies [94,151]) but have received relatively little attention by cichlid researchers.

Finally, some colour variations may have nothing to do with divergent sexual selection, at least initially. Colours may be recruited as arbitrary markers of adaptation to specific ecological conditions, without a mechanistic link between the adaptive trait and the colour signal. For example, the blue and yellow phenotypes of Lake Malawi cichlids may facilitate ecology-assortative mating, even though specific hues may not be consistently linked to specific environmental conditions. At the same time, intraspecific variation in species-specific colouration traits may reveal individual variation in fitness. For example, adaptation to different parasite communities may not only promote the divergence of sexual signals and preferences, but may also expose individual variation by condition-dependent expression of colouration that may affect intraspecific mate choice [152].

The evolution of the haplochromine blotch polymorphism is difficult to characterise as either ecological or arbitrary. Blotch likely influences predation risk, and through linkage with sex determining loci has major effects for individual fitness (see above). Yet, its initial invasion and establishment may be largely driven by stochastic processes such as sex-ratio selection in small populations [27,153]. Similarly, the gold polymorphism in Midas cichlids is not entirely arbitrary, but may evolve largely through stochastic rather than ecologically deterministic mechanisms.

While there is no doubt that colour-mediated mate choice contributes to cichlid speciation, other factors (such as ecological opportunity and geographic structure) are important as well, and may interact with sexual selection. The relative importance of these mechanisms may vary over evolutionary time and differ between lineages [132,154].

## 8 Intrasexual competition: territorial interactions

In several cichlid species, male colour patterns have intimidating effects during aggressive interactions. For example, red colour elements increase an individual's chances of winning territorial contests in the Central American firemouth cichlid (*Thorichthys meeki* [155]) as well as the Lake Victoria haplochromine *Pundamilia nyererei* [156]. In both cases, control experiments under green light, in which red colouration is not visible, reduced the competitive advantage, indicating that it is driven by the opponents’ perception of colour rather than intrinsic differences in aggressive behaviour. In the Midas cichlid, dominance of gold over normal phenotypes may be due to a similar intimidating effect [157,158]. Associations between colouration and aggression are not limited to males. In *Astatotilapia burtoni*, dominant females express male-like colouration [159]. In *Neochromis omnicaeruleus*, females exhibit similar aggression biases as males do [160], and blotched females tend to be more aggressive and dominant than brown females [78]. In convict cichlids, *Cichlasoma nigrofasciatum*, female-specific orange ventral colouration elicits aggressive behaviour in females but not males [161], and in *Pelvicachromis taeniatus*, females with bright ventral colouration are dominant over dull ones [162].

In many vertebrate taxa, the melanocortin system influences both melanin-based colouration and aggressive behaviour [163]. Only one study to date has investigated involvement of the *mc1r* locus in cichlid colouration: in the Midas cichlid, Henning et al. [60] found significant upregulation in the gold morph, perhaps related to its behavioural dominance. Dijkstra et al. [78] speculated that the same gene may be involved in the Lake Victoria haplochromine blotch polymorphism, although the Malawi blotch phenotype has been linked to the *pax7* locus [43,53]. Clearly, additional research in this area is warranted. In addition, it has been suggested that the expression of red and yellow colouration represents a specific physiological trade-off, as it requires carotenoid resources otherwise used for antioxidant function [79,164] (but see [165]).

### 8.1 Colouration-mediated aggression and speciation

In recent years, the important role of cichlid colouration in both intra- and interspecific aggression has inspired a series of studies into its effects on species divergence and coexistence. Lorenz [166] already suggested that colour differences between coral reef fishes could contribute to species coexistence, by facilitating targeted aggression towards those individuals most likely to compete for the same resources (such as food, territories and mates). Consistent with this idea, observations on African Lake cichlids have shown that males tend to be more aggressive towards conspecific than towards heterospecific males, and that neighbouring territories are often occupied by males of different species [167–170]. Confirming the critical role of colouration, Seehausen and Schluter [171] documented that among closely related species in Lake Victoria, males of differently coloured species are more likely to occupy adjacent territories than males of species that are similarly coloured. They suggested that male-male competition exerts negative frequency-dependent selection on colouration, thereby promoting colour diversification.

**Fig. 6:**
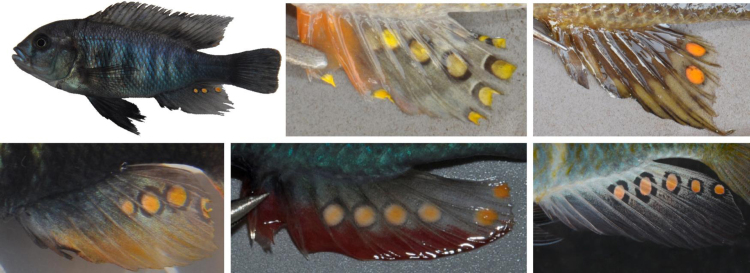
Egg spots on the anal fins of haplochromine males. Top row: *Neochromis omnicaeruleus* (Lake Victoria), *Interochromis loockii* and *Petrochromis ephippium* (Lake Tanganyika); bottom row: *Pundamilia nyererei* (Lake Victoria), *Astatotilapia calliptera* (Lake Malawi), *A. burtoni* (rift valley rivers). Photographs by Tania Bosia (Tanganyika and Malawi species), Anya Theis (*A. burtoni*) and Oliver Selz (Victoria species).

Dijkstra et al. [172] addressed these ideas experimentally, using different populations of the Lake Victoria species pair *Pundamilia pundamilia* (blue males) and *P. nyererei* (red males; Fig. 5). They found that blue males from populations in which the red species is absent were more aggressive towards blue males than towards red males, suggesting that novel colour morphs would benefit from reduced aggression, facilitating their establishment. A similar pattern was found for populations where blues and reds are reproductively isolated, but in hybridising populations they found increased aggression towards red males. Possibly, competition for mating opportunities explains this pattern: only in hybridising populations do males compete for the same females. Pauers et al. [173] obtained similar results in the Lake Malawi cichlid *Metriaclima mbenjii*. By using two differently coloured species as heterospecific stimulus males, they confirmed that the observed aggression bias for conspecifics was mediated by colour differences: focal males performed more aggressive display towards both conspecifics and similarly coloured heterospecifics, as compared to differently coloured heterospecifics. Observations in *Astatotilapia burtoni* may be consistent with the idea that competition over mates underlies male aggression biases. Blue and yellow morphs, that represent flexible phenotypes that are not reproductively isolated [77], did not bias their aggression towards similarly coloured rivals [74]. Together, the data indicate that while aggression biases may facilitate the initial establishment of novel colour phenotypes, and promote coexistence among reproductively isolated species, they may not contribute to the coexistence of hybridising morphs (see Fig. 6).

## 9 Concluding remarks

Decades of research on the diverse colouration patterns of cichlid fishes have identified a multitude of selective pressures involved in their origin and divergence. A major challenge for future work is to establish how developmental and genetic mechanisms are related to evolutionary patterns: do similar phenotypes emerge from shared developmental pathways, and to what extent do such pathways lead to predictable patterns of variationtd:quest Answering these questions will be important for evaluating the relative importance of developmental biases and constraints on the one hand, and extrinsic ecological conditions on the other, for explaining the taxonomic and geographic distribution of (cichlid) colour variation. Our review of the literature is certainly not complete, but probably illustrates a real bias in research effort: not only are some taxa studied much more intensively than others, we also see that different hypotheses are tested, and different methods applied, in different lineages. We expect that in the next decades, research on cichlid colours will become more integrative. The genomics revolution allows both deeper and broader understanding of the mechanisms underlying colour variation, and these insights will be particularly informative when accompanied by ecological and behavioural studies. In this way, we can take full advantage of the cichlid model system as a tool for understanding the evolution of biological diversity.

## Figures and Tables

Figure from Elmer et al. [7].

Photos by Oliver Selz.

**Table 1:** An overview of experimental studies on the role of colouration in assortative mating among species and allopatric populations of cichlid fishes.

Taxa	Origin	Sym-/allopatric	Conditions	Preferences	Reference
Visual cues only					
*Pseudotropheus* (*Metriaclima*) zebra complex	Lake Malawi	Sympatric	Broad spectrum light	Positive assortative	[184]
*Labeotropheus*	Lake Malawi	Allopatric	Broad spectrum light	Positive assortative	[185,186]
*Pseudotropheus* (*Metriaclima*) zebra complex	Lake Malawi	td:quest	Broad spectrum light	Positive assortative; when conspecific males were not available, then colour-dependent preference for most similar species	[187]
*Pseudotropheus* (*Metriaclima*) zebra complex	Lake Malawi	Sympatric	Broad spectrum light	Positive assortative	[188]
			Monochromatic light	Positive assortative	
*Pundamilia*	Lake Victoria	Sympatric	Broad spectrum light	Positive assortative	[189]
			Monochromatic light	Random	
*Pseudotropheus* (*Metriaclima*) zebra complex	Lake Malawi	Allopatric	Broad spectrum light	Random	[113]
*Pseudotropheus* (*Metriaclima*) zebra complex	Lake Malawi	Sympatric	Broad spectrum light	Random	[114]
*Pseudotropheus* (*Metriaclima*) zebra complex and *Cynotilapia*	Lake Malawi	td:quest	Broad spectrum light	Dependent on colour pattern similarity, but not strictly positive assortative	[106]
*Apistogramma*	South America	Allopatric	Broad spectrum light	Dependent on colour pattern similarity	[108]
Visual and/or other cues					
*Pseudotropheus* (*Metriaclima*) zebra complex	Lake Malawi	Sympatric	Full contact, or visual + olfactory	Positive assortative	[114]
*Pseudotropheus* (*Metriaclima*) zebra complex	Lake Malawi	Allopatric	Full contact	Positive assortative to random; no discrimination between two similarly coloured morphs	[107]
*Pseudotropheus* (*Metriaclima*) zebra complex	Lake Malawi	Sympatric	Full contact	Positive assortative	[190]
*Pseudotropheus* (*Metriaclima*) zebra complex	Lake Malawi	Allopatric	Full contact	Random to complete; independent of colour similarity	[113]
*Pseudotropheus* (*Metriaclima*) zebra complex	Lake Malawi	Allopatric	Full contact under monochromatic light	Positive assortative in one species, random in another	[113]
*Tropheus*	Lake Tanganyika	Allopatric	Full contact	Positive assortative to random; not always correlated with colour pattern similarity	[109,191,192]
*Apistogramma*	South America	Allopatric	Full contact	Positive assortative	[121]
*Pseudotropheus* (*Metriaclima*) zebra complex	Lake Malawi	Sympatric	Olfactory only	Random	[188]

## Box 1 Egg dummies

One colour pattern that is characteristic for the mouthbrooding haplochromine cichlids are the yellow or orange spots on the anal fins. These occur in the majority of haplochromine species and in both sexes, but are more pronounced, often surrounded by a characteristic transparent ring, in males Fig. 6. Their occurrence in mouthbrooders, together with the striking resemblance to haplochromine eggs, inspired the hypothesis that these spots are functional in the spawning context: females often peck at these spots during spawning, presumably mistaking the spots for eggs, and thereby picking up sperm resulting in increased fertilisation success and introducing strong selection on this trait in males [174,175]. However, experimental data do not support this idea: eggspot removal did not reduce fertilisation rates in *Astatotilapia elegans* [175] and *A. burtoni* [176], nor did natural variation in eggspot numbers (*Pseudotropheus aurora* [177]). Several alternative hypotheses for the evolution of eggspots have been investigated. In some species, eggspot characteristics may be subject to sexual selection by female choice (e.g. *A. elegans* [175]; *Pseudotropheus aurora* [177]; *P. lombardi* [178]), but in other species this is not the case (*A. burtoni* [176,179]). Interestingly, female *Pseudocrenilabrus multicolor* prefer males with spots, even though their own males don’t have them, possibly indicating an ancestral sensory bias [180]. Finally, there is some evidence that eggspots may have an intimidating effect in aggressive interactions in *A. burtoni* [176,179]. Goldschmidt [181] suggested that divergence in eggspot characteristics, perhaps driven by environmental influences on egg colour, size or shape, could promote reproductive isolation. Compared to other colour patterns, however, egg spots are much less diverse between species. A recent analysis of egg spot colouration in two *Pundamilia* species revealed parallel changes across different light environments, suggesting that eggspots do indeed adapt to ecological conditions but do not contribute to species differentiation [23].

Egg-mimicking colour patches may not be restricted to haplochromines. Some sister lineages exhibit red or yellow anal fin markings, that may or may not be considered egg dummies [180,182]. In some Ectodini, males have yellow tips on their elongated pelvic fins that might mimic eggs [183], although their function has not been investigated.
